# Paracetamol in Pregnancy: Uncertain Evidence, Certain Consequences

**DOI:** 10.5694/mja2.70203

**Published:** 2026-05-14

**Authors:** David J. Tunnicliffe, Miranda Cumpston, Debra Kennedy, Margie Danchin, Armando Teixeira‐Pinto

**Affiliations:** ^1^ School of Public Health The University of Sydney Sydney New South Wales Australia; ^2^ Centre for Kidney Research Children's Hospital at Westmead Westmead New South Wales Australia; ^3^ School of Public Health and Preventative Medicine Monash University Melbourne Victoria Australia; ^4^ MotherSafe Royal Hospital for Women Randwick New South Wales Australia; ^5^ School of Women's and Children's Health University of New South Wales Sydney New South Wales Australia; ^6^ Department of Paediatrics The University of Melbourne Melbourne Victoria Australia; ^7^ Vaccine Clinical Trials and Uptake Group Murdoch Children's Research Institute Parkville Victoria Australia

**Keywords:** epidemiology, guidelines as topic, health communication, pain, pregnancy, public health, systematic review

## Abstract

Autism diagnoses have increased over the past decade, prompting debate on potential causes. In September 2025, US President Donald Trump claimed that paracetamol is a ‘big factor’ in autism, citing a systematic review based solely on observational studies. The review's selective reporting, methodological flaws (including applying an environmental health rather than evidence‐based medicine framework) and lack of causal evidence provided weak justifications for its conclusions and have fuelled public confusion about paracetamol safety in pregnancy. This article critically appraises the review and examines how scientific uncertainty can be manipulated and amplified within broader public health domains.

## 1 Introduction

On 22 September 2025, US President Donald Trump and senior health officials linked acetaminophen (paracetamol) use in pregnancy to rising autism diagnoses [1]. However, high quality studies do not support a causal link between paracetamol and neurodevelopmental disorders [2, 3]. The announcement spread globally, creating confusion about a widely used medicine and illustrating how scientific uncertainty can be amplified in ways that can erode trust in public health advice.

The claims drew on a systematic review by Prada and colleagues 2025 [4] (hereafter, the Prada review), which synthesised findings from 46 studies using the Navigation Guide, an environmental health framework that integrates human and non‐human evidence [5]. While appropriate in regulatory contexts, such frameworks differ fundamentally from evidence‐based medicine approaches designed to guide public health and clinical decision‐making. The Prada review emphasised studies reporting an association between prenatal paracetamol exposure and both attention‐deficit/hyperactivity disorders (ADHD) and autism [6], while downplaying contradictory findings, including a large Swedish cohort study in which associations attenuated under sibling controls [7]. Health professionals responded swiftly to the Trump announcement, noting the lack of randomised evidence and the importance of findings from sibling‐controlled designs that account for shared familial and genetic confounding.

Importantly, the significance of this debate extends beyond paracetamol safety in pregnancy. Selective use of studies in political and media discourse can shape perceptions of partisanship. In this case, expert concerns were met with counter‐accusation of bias [8], shifting attention away from the methodological limitations of the Prada review to perceived political alignment. Similar dynamics have been observed across multiple contemporary public health domains, including vaccination, environmental exposure and pandemic response, where uncertainty is selectively framed to reinforce narratives, polarise debate and influence public perception and behaviour.

Subsequent high‐quality systematic reviews conducted to evidence‐based medicine standards have since clarified that the available evidence does not support a causal association between prenatal paracetamol exposure and neurodevelopmental disorders [9, 10]. The processes by which uncertainty was generated, amplified and communicated remain highly relevant. The Prada review provides a useful case study for examining how methodological choices, departures from established evidence‐based medicine standards and narrative handling of uncertainty can confer unwarranted epistemic authority when scientific findings intersect with politicised health messaging.

Therefore, we critically appraise the methodological quality of the Prada review using evidence‐based medicine standards, not simply to reassess its conclusions, but to illustrate how scientific uncertainty can be amplified beyond the literature, with implications for clinical practice and health communication.

## 2 Scientific Assessment of the Prada Review

The Navigation Guide is a less established method for synthesising and appraising evidence. Although typically used for environmental exposures [11], and designed to combine human and non‐human studies, the Prada review included only human observational data, despite available animal studies [12, 13]. More established and developed methodologies for synthesising observational data (Supporting Information: Section 1) are used by bodies such as Cochrane and the World Health Organization. We assessed the conduct of the Prada review using AMSTAR 2 (A MeaSurement Tool to Assess systematic Reviews, version 2) [14] and ROBIS (Risk of Bias in Systematic Reviews) [15] (Table 1 and Supporting Information: Section 2).

**TABLE 1:** A Measurement Tool to Assess Systematic Reviews Version 2 (AMSTAR 2) checklist [14] of Prada et al. 2025 [4].

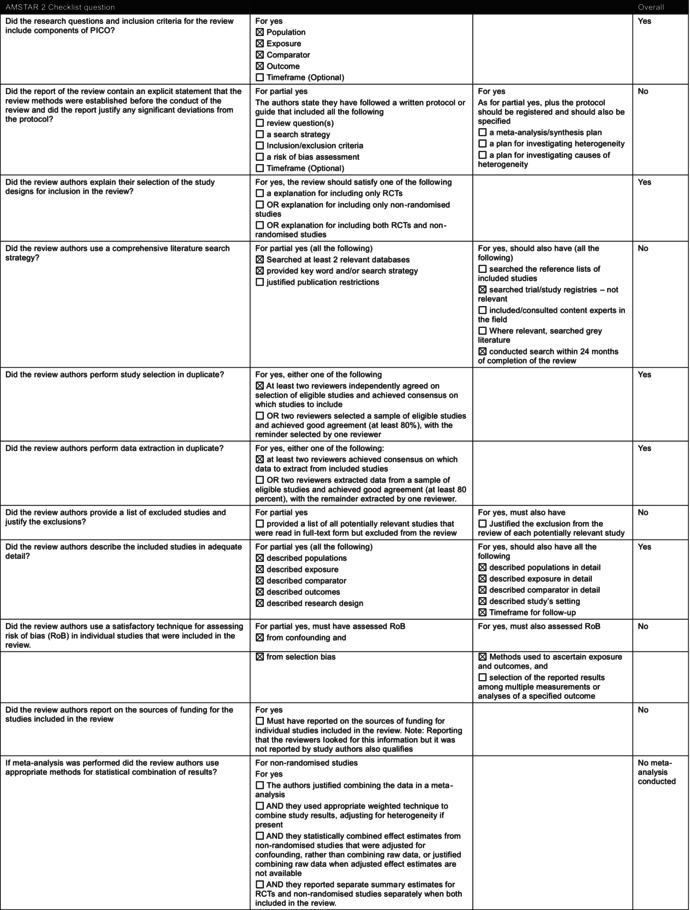 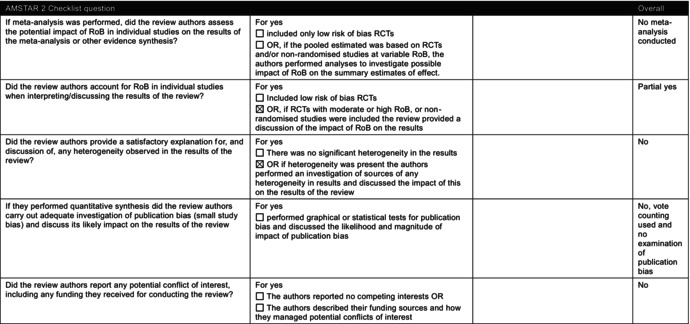

Abbreviations: PICO, Population, Intervention, Comparator, Outcome; RCT, randomised controlled trial; RoB, risk of bias.

### 2.1 Critical Appraisal With Evidence‐Based Medicine Standards

#### 2.1.1 Methods Prespecification and Study Selection

The Prada review did not prespecify its methods, and no protocol was registered or published. Its search strategy lacked sufficient detail for replication, unpublished studies were not sought and no excluded studies list was provided. The senior author, Andrea Baccarelli, Dean of the Harvard TH Chan School of Public Health, declared a conflict as an expert witness in litigation concerning paracetamol and neurodevelopmental disorders. But the review did not explain how this conflict was managed. The case was later dismissed for insufficient evidence. The judge from this case also noted that his testimony failed to acknowledge the ‘role of genetics in aetiology’ [16] for autism. Without transparent study selection and clear management of declared conflicts, readers cannot be confident that the review's conclusions are not shaped by selective inclusion of studies, or investigator bias rather than the totality of the available evidence.

#### 2.1.2 Risk of Bias Assessment

The methods used to assess risk of bias were not consistent with current standards and offered minimal justifications. However, some domains, such as confounding, were addressed by the reviewers, that is, adjustment for key variables, use of propensity score matching and evaluation of residual confounding. Domain‐level assessments were converted to numerical scores and averaged, a practice widely discouraged [17] because it can mask domains with critical risk of bias. In four autism studies, averaging suggested low risk of bias despite some domains being rated as critical. These departures from established risk of bias assessment can obscure serious methodological flaws in studies, giving a misleading impression of the overall strength of evidence.

#### 2.1.3 Synthesis of Studies

A meta‐analysis was not conducted due to the diversity of the included studies, in terms of their populations, methods of assessing paracetamol use and time frames of recall. This decision is not unreasonable; a meta‐analysis of observational studies is challenging, and can yield misleading summary estimates [18]. However, the Prada review uses vote counting based on the statistical significance of study results, classifying studies as showing a positive association, null association or mixed, without a clear definition of ‘mixed’. Vote counting based on statistical significance is widely considered inappropriate [17, 19], and much the same diversity that precluded a meta‐analysis should also prevent the use of vote counting to infer an overall direction of effect across the included studies. Although sources of heterogeneity were identified (e.g., maternal self‐reports vs. biomarkers, timing and duration of exposure, outcome measures and confounder adjustment approaches), their impact was not systematically assessed. Instead, heterogeneity was discussed narratively, creating scope for selective emphasis that can align with prior assumptions. In the absence of a structured heterogeneity analysis, such discussions risk serving as post hoc justification and limit confidence in any inferred direction or magnitude of effect.

#### 2.1.4 Certainty of the Evidence

The Prada review reported using GRADE (Grading of Recommendations Assessment, Development and Evaluation) to assess the certainty of the evidence, but deviated substantially from published guidance, applying selective domains and assessing individual studies with a non‐standard scoring system [19]. Studies were rated from very strong to very weak based on criteria partly drawn from GRADE components, such as large relative effect and dose–response. These ratings were summed into a ‘strength of evidence’ score with little clarity on derivation. The use of a non‐standard certainty of the evidence rating without transparent justifications may overstate confidence in the overall findings of the review. Furthermore, the authors removed the lowest‐scoring studies, and an unclear ‘expert opinion score’ was added, but its purpose and calculation were not well explained. The authors appear to have reweighted criteria based on their assessment of study flaws, blurring the distinction between evidence appraisal and expert opinion.

#### 2.1.5 Risk of Bias in Interpretation

**FIGURE 1:**
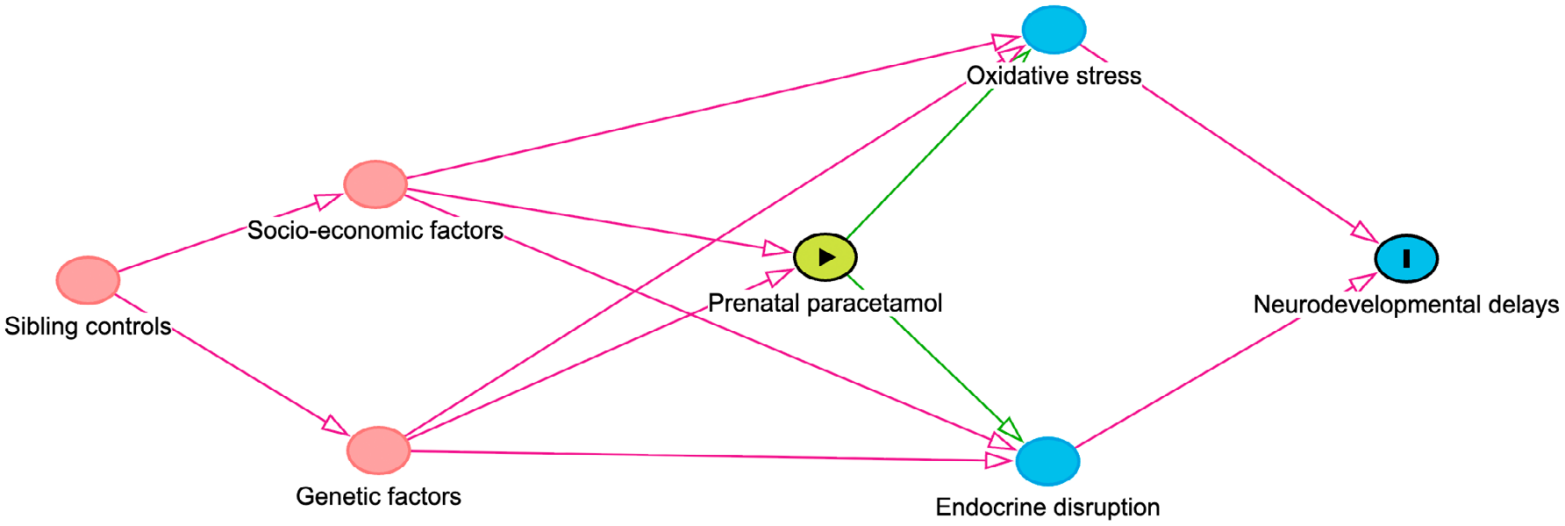
Directed acyclic graph illustrating how the Prada et al. review [4] hypothesised relationships between prenatal paracetamol exposure, neurodevelopment outcomes, shared familial factors and proposed biological mediators (oxidative stress and endocrine disruption). Sibling‐controlled designs account for shared familial factors. Arrows represent hypothesised causal relationships.

The review's discussion focused heavily on criticising the two sibling‐matched studies [7]. The Swedish study [7] was rated with a high risk of bias due to a lack of specificity in measuring paracetamol exposure, relying on routinely collected midwife interviews without questions on paracetamol use, resulting in presumed underreporting (~7.5%) of paracetamol use in pregnancy versus other studies (~50%) [20]. Most studies relied on maternal self‐report, introducing potential recall bias; only a few used biomarkers, such as paracetamol concentrations in core blood or the meconium. Prada and colleagues argued that sibling controls may introduce bias by relying on discordant pairs for exposure and outcome, reducing statistical power. However, the Swedish study [7] retained over 31,000 participants from the initial 2.48 million, mitigating this concern. Sibling controls may cause non‐differential exposure misclassification if recall is similar across pregnancies for siblings. Prospectively collected midwife data may reduce this risk. While sibling controls address socio‐economic status and genetic confounding, they may also adjust for shared mediators. Sibling controls may also attenuate associations if biological mechanisms (e.g., increased oxidative stress and endocrine disruptions) are partly shared within families. In such cases, shared components may be removed, potentially obscuring true effects (Figure 1). However, this depends on the underlying causal pathway, which has not been established.

The authors of the Prada review concluded that there was ‘strong evidence of a likely association between prenatal paracetamol use and increased risk of ADHD, autism and neurodevelopment delays in children’. They argued that triangulation with earlier reviews [21, 22] strengthened their conclusions, although those reviews pre‐dated the Swedish study [7, 23]. Prada and colleagues acknowledged possible residual confounding but argued that their evaluation of exposure assessment, the use of variable adjustments and negative controls made confounding unlikely. The authors advised judicious paracetamol use in pregnancy but acknowledged its favourable safety profile compared with other analgesics, such as non‐steroidal anti‐inflammatory drugs.

## 3 Conclusions

Our assessment identified major methodological issues in the Prada review [4] that limit confidence in its conclusions. Readers cannot be assured that all relevant studies were included or that risk of bias, certainty in the evidence or between‐study differences were appropriately assessed. Reviews that do not meet systematic review standards should not guide practice. Although the Prada review raises valid concerns regarding sibling controls, the extent to which these concerns meaningfully affect causal interpretation remains unclear.

More recent umbrella and systematic reviews [9, 10] have since clarified that associations between paracetamol and neurodevelopmental outcomes are attenuated once key sources of bias are addressed. These reviews consistently highlight residual confounding, including maternal infection, inflammation or fever, which both prompt paracetamol use and independently influence neurodevelopment. Importantly, these analyses were conducted using established evidence‐based medicine standards, including structured assessment of risk of bias, confounding, heterogeneity assessment and evaluating the certainty of the evidence.

The contrast between these reviews and the Prada review underscores the importance of systematic review methods. The Prada review was misleading, not only because of its conclusions, but because the methodological choices allowed for presentation of findings with authority disproportionate to the underlying certainty of the evidence. The more recent reviews [9, 10] contextualised uncertainty, highlighted the limitations of observational data and resisted causal claims with insufficient evidence. This comparison illustrates how different methodological choices and different narrative handling of uncertainty can shape scientific and public understanding.

The downstream consequences of such misinterpretation can be substantial. Incorrect or selectively framed health information from political and regulatory leaders risks harm and erodes public trust. Statements by the Trump Administration and Federal Drug Administration may prompt pregnant women to avoid treating fever or select less safe alternatives, despite paracetamol remaining the safest option in pregnancy, while others may increase use unnecessarily. Untreated fever itself poses risks, and other painkillers carry the risk of fetal harms. Families may also experience anxiety about past exposures, which may adversely affect the mother–child relationship. These reactions demonstrate the tangible consequences of politicised health communication and underscore the importance of consistent messaging from trusted sources. Misinformation, whether deliberate or unintentional, can exploit structural weaknesses in our institutions and health systems. Clinical and public health recommendations should follow established processes, like evidence‐to‐decision frameworks [19], which weigh benefits and harms, values, acceptability, equity and resource implications.

Although Australia is less polarised than the United States, similar risks exist. After the Trump announcement, Australian institutions responded swiftly, with bodies such as the Australian Academy of Health and Medical Research [2] and Royal Australian and New Zealand College of Obstetricians and Gynaecologists [3] providing reassurance about paracetamol safety in pregnancy. Continued reliance on these organisations is essential, but addressing the current gap will require more formalised and stronger mechanisms. Strengthening rapid‐response evidence‐appraisal infrastructure, modelled on the National Clinical Evidence Taskforce, would support timely, rapid evidence updates. Improved coordination of national and state health communications to ensure consistent messaging would help clinicians access clear guidance and counter misinformation. The success of Australia's coronavirus disease living guidelines demonstrates the feasibility and value of such structures [24]. Similar proactive approaches will be crucial in maintaining public confidence in evidence‐based healthcare.

## Author Contributions

**David J. Tunnicliffe:** conceptualisation, formal analysis, and writing of the original draft, and reviewing and editing the manuscript. **Miranda Cumpston:** formal analysis, writing and editing the manuscript. **Debra Kennedy:** writing and editing the manuscript. **Margie Danchin:** writing and reviewing the manuscript. **Armando Teixeira‐Pinto:** conceptualisation, reviewing the editing of the manuscript.

## Funding

David J. Tunnicliffe is supported by an National Health and Medical Research Council Emerging Leadership 1 Investigator Grant (APP1197337) and University of Sydney, Faculty of Medicine and Health Talented Researcher Scheme. The funding organisations had no role in the design and conduct or approval of the manuscript.

## Disclosure

Not commissioned; externally peer reviewed.

## Conflicts of Interest

The authors declare no conflicts of interest.

## Supporting information


**Data S1:** mja270203‐sup‐0001‐supinfo.pdf.

## Data Availability

No new datasets were generated or analysed in this study. This work appraises a previously published systematic review, and all data can be accessed through the original article as cited.
