# Supplementary material for: Paracetamol in Pregnancy: Uncertain Evidence, Certain Consequences
Source: Med J Aust. 2026 May 14;224:e70203. doi: 10.5694/mja2.70203 (PMC13175944; doi:10.5694/mja2.70203)
Supplement: Supplementary file 1 — Data S1: mja270203‐sup‐0001‐supinfo.pdf. [file MJA2-224-0-s001.pdf]

Supporting Information

Supplementary material

This appendix was part of the submitted manuscript and has been peer reviewed. It is posted as supplied by the authors.

Appendix to: Tunnicliffe DJ, Cumpston M, Kennedy D, Danchin M, Teixeira-Pinto A. Paracetamol in Pregnancy: Uncertain Evidence, Certain Consequences. *Med J Aust* 2026; doi: 10.5694/mja2.70203.

Section 1: Evidence-Based Medicine Abbreviations

Abbreviation	Full-term	Brief description	Reference
PRISMA	Preferred Reporting Items for Systematic Reviews and Meta-Analyses	Reporting guideline for systematic reviews.	Page MJ, McKenzie JE, Bossuyt PM, Boutron I, Hoffmann TC, Mulrow CD, et al. The PRISMA 2020 statement: an updated guideline for reporting systematic reviews. BMJ. 2021;372:n71.
SWiM	Synthesis Without Meta-analysis	Guidance for narratively synthesising studies.	Campbell M, McKenzie JE, Sowden A, Katikireddi SV, Brennan SE, Ellis S, et al. Synthesis without meta-analysis (SWiM) in systematic reviews: reporting guideline. BMJ. 2020;368:l6890.
ROBINS-E	Risk of Bias in Non-randomised Studies of Exposures	Tool for assessing bias in exposure-related observational studies.	Higgins JPT, Morgan RL, Rooney AA, Taylor KW, Thayer KA, Silva RA, et al. A tool to assess risk of bias in non-randomized follow-up studies of exposure effects (ROBINS-E). Environ Int. 2024;186:108602.
AMSTAR-2	A Measurement Tool to Assess Systematic Reviews, Version 2	Appraisal tool for systematic reviews of healthcare interventions.	Shea BJ, Reeves BC, Wells G, Thuku M, Hamel C, Moran J, et al. AMSTAR 2: a critical appraisal tool for systematic reviews that include randomised or non-randomised studies of healthcare interventions, or both. BMJ. 2017;358:j4008.
ROBIS	Risk of Bias in Systematic Reviews	Tool for evaluating risk of bias within systematic reviews.	Whiting P, Savović J, Higgins JP, Caldwell DM, Reeves BC, Shea B, et al. ROBIS: A new tool to assess risk of bias in systematic reviews was developed. J Clin Epidemiol. 2016;69:225-34.
GRADE	Grading of Recommendations, Assessment, Development and Evaluation	Framework for judging certainty in evidence and strength of recommendations.	Senerth E, Whaley P, Akl E, Beverly B, Alonso-Coello P, Rooney A, et al. GRADE guidance 40: The GRADE evidence-to-decision framework for environmental and occupational health. Environ Int. 2025;197:109314.

Section 2: Risk of Bias in Systematic Reviews (ROBIS)

Study Details

Prada D, Ritz B, Bauer AZ, Baccarelli AA. Evaluation of the evidence on acetaminophen use and neurodevelopmental disorders using the Navigation Guide methodology. Environ Health. 2025;24(1):56.

Phase 1: Assessing relevance (Optional):

Intervention reviews:

Category	Target question (e.g. overview or guideline)	Review being assessed
Patients/Population(s):	Pregnant women	Pregnant women
Intervention(s):	Paracetamol	Paracetamol
Comparator(s):	No paracetamol	No paracetamol
Outcome(s):	Autism, ADHD	Autism & neurodevelopmental outcomes

Does the question addressed by the review match the target question?	yes

Phase 2: Identifying concerns with the review process

DOMAIN 1: STUDY ELIGIBILITY CRITERIA	
Describe the study eligibility criteria, any restrictions on eligibility and whether there was evidence that objectives and eligibility criteria were pre-specified:	
1.1 Did the review adhere to pre-defined objectives and eligibility criteria?	Probably yes
1.2 Were the eligibility criteria appropriate for the review question?	Yes
1.3 Were eligibility criteria unambiguous?	Yes
1.4 Were any restrictions in eligibility criteria based on study characteristics appropriate (e.g. date, sample size, study quality, outcomes measured)?	Yes
1.5 Were any restrictions in eligibility criteria based on sources of information appropriate (e.g. publication status or format, language, availability of data)?	Yes
Concerns regarding specification of study eligibility criteria	Low
Rationale for concern:	

DOMAIN 2: IDENTIFICATION AND SELECTION OF STUDIES	
Describe methods of study identification and selection (e.g. number of reviewers involved):	
2.1 Did the search include an appropriate range of databases/electronic sources for published and unpublished reports?	Yes
2.2 Were methods additional to database searching used to identify relevant reports?	No
2.3 Were the terms and structure of the search strategy likely to retrieve as many eligible studies as possible?	No
2.4 Were restrictions based on date, publication format, or language appropriate?	Yes
2.5 Were efforts made to minimise error in selection of studies?	Yes
Concerns regarding methods used to identify and/or select studies	High
Rationale for concern:	Sources other than major databases were not searched. Search

DOMAIN 2: IDENTIFICATION AND SELECTION OF STUDIES	
	terms used were very limited.

DOMAIN 3: DATA COLLECTION AND STUDY APPRAISAL	
Describe methods of data collection, what data were extracted from studies or collected through other means, how risk of bias was assessed (e.g. number of reviewers involved) and the tool used to assess risk of bias:	
3.1 Were efforts made to minimise error in data collection?	Yes
3.2 Were sufficient study characteristics available for both review authors and readers to be able to interpret the results?	Yes
3.3 Were all relevant study results collected for use in the synthesis?	Probably not
3.4 Was risk of bias (or methodological quality) formally assessed using appropriate criteria?	No
3.5 Were efforts made to minimise error in risk of bias assessment?	No information
Concerns regarding methods used to collect data and appraise studies	High
Rationale for concern:	The process for selection of study results for inclusion in the review is not provided and reporting of study results is limited.<br>Risk of bias did not use an appropriate assessment method.

DOMAIN 4: SYNTHESIS AND FINDINGS	
Describe synthesis methods:	
4.1 Did the synthesis include all studies that it should?	Yes
4.2 Were all pre-defined analyses reported or departures explained?	No
4.3 Was the synthesis appropriate given the nature and similarity in the research questions, study designs and outcomes across included studies?	No
4.4 Was between-study variation (heterogeneity) minimal or addressed in the synthesis?	No
4.5 Were the findings robust, e.g. as demonstrated through funnel plot or sensitivity analyses?	No information
4.6 Were biases in primary studies minimal or addressed in the synthesis?	No
Concerns regarding the synthesis and findings	High
Rationale for concern:	Prespecified analyses were not described.<br>Synthesis method was vote counting based on statistical significance, which is not appropriate.<br>Identified sources of heterogeneity (e.g., maternal self-reports vs. biomarkers, timing and

DOMAIN 4: SYNTHESIS AND FINDINGS	
	duration of exposure, outcome measures and confounder adjustment approaches were not reported across studies or systematically assessed.

Y=YES, PY=PROBABLY YES, PN=PROBABLY NO, N=NO, NI=NO INFORMATION

Phase 3: Judging risk of bias

Summarize the concerns identified during the Phase 2 assessment:

**Domain**	**Concern**	**Rationale for concern**
1. Concerns regarding specification of study eligibility criteria	Low	
2. Concerns regarding methods used to identify and/or select studies	High	Sources other than major databases were not searched. Search terms used were very limited.
3. Concerns regarding methods used to collect data and appraise studies	High	The process for selection of study results for inclusion in the review is not provided and reporting of study results is limited.<br>Risk of bias did not use an appropriate assessment method.
4. Concerns regarding the synthesis and findings	High	Prespecified analyses were not described. Synthesis method was vote counting based on statistical significance, which is not appropriate.<br>Identified sources of heterogeneity (e.g., maternal self-reports vs. biomarkers, timing and duration of exposure, outcome measures and confounder adjustment approaches were not reported across studies or systematically assessed.

RISK OF BIAS IN THE REVIEW	
Describe whether conclusions were supported by the evidence:	
A. Did the interpretation of findings address all of the concerns identified in Domains 1 to 4?	No
B. Was the relevance of identified studies to the review's research question appropriately considered?	Yes
C. Did the reviewers avoid emphasizing results on the basis of their statistical significance?	No
Risk of bias in the review	High

RISK OF BIAS IN THE REVIEW	
Rationale for risk:	Search not sufficiently comprehensive. Results reporting was limited. Risk of bias did not use an appropriate method. GRADE was misapplied to modify strength of conclusions. Heterogeneity not systematically investigated. Conclusions based on vote counting based on statistical significance.

Y=YES, PY=PROBABLY YES, PN=PROBABLY NO, N=NO, NI=NO INFORMATION
